# CACTI: Free, Open-Source Software for the Sequential Coding of Behavioral Interactions

**DOI:** 10.1371/journal.pone.0039740

**Published:** 2012-07-16

**Authors:** Lisa H. Glynn, Kevin A. Hallgren, Jon M. Houck, Theresa B. Moyers

**Affiliations:** Department of Psychology, University of New Mexico, Albuquerque, New Mexico, United States of America; Imperial College London, United Kingdom

## Abstract

The sequential analysis of client and clinician speech in psychotherapy sessions can help to identify and characterize potential mechanisms of treatment and behavior change. Previous studies required coding systems that were time-consuming, expensive, and error-prone. Existing software can be expensive and inflexible, and furthermore, no single package allows for pre-parsing, sequential coding, and assignment of global ratings. We developed a free, open-source, and adaptable program to meet these needs: The CASAA Application for Coding Treatment Interactions (CACTI). Without transcripts, CACTI facilitates the real-time sequential coding of behavioral interactions using WAV-format audio files. Most elements of the interface are user-modifiable through a simple XML file, and can be further adapted using Java through the terms of the GNU Public License. Coding with this software yields interrater reliabilities comparable to previous methods, but at greatly reduced time and expense. CACTI is a flexible research tool that can simplify psychotherapy process research, and has the potential to contribute to the improvement of treatment content and delivery.

## Introduction

The objective, systematic study of communication has been an essential element of psychotherapy process research for over seven decades [1]. This approach to studying the interactions between clients and clinicians within treatment sessions spread rapidly with the introduction of inexpensive analog audio recording equipment [2], a technology that permitted the preservation and study of these interactions. The behavior coding approach applies standardized ratings systems of clinician and client speech to these rich data sets. By counting occurrences of specific verbal behaviors, or rating the intensity of such behaviors, the rater permits the assessment of the effects of within-session behaviors on outcome (e.g., [3]), the impact of clinician training (e.g., [4]), the evaluation of clinician adherence to or competence in specific therapeutic approaches (e.g., [5]), and, most recently, the exploration of theoretically derived mechanisms of behavior change (e.g., [6]).

Behavior coding also has been applied to capture the sequential dependencies between behaviors [7], [8], [9], [10], [11]. By preserving the temporal sequence of behaviors, the sequential approach permits the analysis of mutual influence between client and clinician, which can help to identify and test hypothesized mechanisms of action of psychotherapies and potentially improve treatment delivery and outcomes [12], [13]. Sequential analyses may range in complexity from classic lag-sequential techniques [7] to hidden Markov models [14].

A simple method of preserving in-session data for sequential analysis is the audio-recording of psychotherapy sessions. Because voice recognition software often cannot reliably discriminate between two or more novel voices in a recording, human transcriptionists generally have been used to transcribe the content of audio-recorded interviews, which then are subject to review using an objective coding system. This process is costly, time-consuming, and often error-prone (e.g., [15]), but historically has been a necessary step in sequential coding. A good example of this methodology is derived from the study of potential mechanisms of change in motivational interviewing ( [16]), an evidence-based psychotherapy for treating substance use disorders and other problematic health behaviors. Until recently, the sequential analysis of motivational interviewing sessions has been accomplished through a tedious, multi-step process (e.g., [10]): Analog audio-recorded sessions were outsourced to professional transcriptionists or transcribed in-house by lab assistants; coders divided and assigned ratings to client and clinician speech by playing back audio recordings and making notations on printed transcripts in pencil; and handwritten data were entered manually into statistical software. Although this method was thorough and ultimately effective, it also was resource-intensive and inefficient.

As computer capabilities have improved and digital audio recorders have become widely available, an array of software packages has emerged to assist in the analysis of audio recordings. At least eleven recent commercial and academic software packages, which range in intended purpose from qualitative analysis to animal-behavior observation, can be used to code behavioral interactions. Those include ATLAS.ti [17], Computer-Aided Protocol Analysis System(CAPAS 2.0) [18], Computerized Profiling (CP) [19], [20], CowLog [21], Dartfish [22], Digital Replay System [23], Noldus Observer Video-Pro [24], Noldus Observer XT [25], Nvivo 9 [26], Observational Data Coding System (ODCS) [27], and Transana [28]. Although these products provide clear benefits over printed transcripts, they also show insurmountable drawbacks for psychotherapy process research, such as being expensive, unmodifiable, or limited in function. Psychotherapy process research would benefit from software that could assist in the moment-to-moment parsing, sequential coding, and global rating of audio-recorded psychotherapy sessions, as well as be easily adaptable to various projects and coding systems and ideally would be freely available. We developed the CASAA Application for Coding Treatment Interactions (CACTI) to meet these demands.

This article introduces CACTI as a flexible and transcript-free program for rating digital audio recordings of psychotherapy sessions and other behavioral interactions. Herein we describe CACTI’s development, system requirements, features, advantages, and known limitations; narrate the features of CACTI; and discuss future directions for the software and its applications for research.

## Methods

### Ethics Statement

The parent study, Project ELICIT, was overseen by the University of New Mexico Main Campus Institutional Review Board. All participants (i.e., substance-abuse treatment providers) provided written informed consent prior to their participation. The present study constituted a re-analysis of existing data, and all coders and software testers were approved as investigators by the aforementioned Institutional Review Board.

### Development of CACTI

CACTI was developed for Project ELICIT, a randomized study of two training methods for motivational interviewing with 190 front-line substance-abuse treatment providers [29]. The software was designed for use with the Motivational Interviewing Skill Code (MISC 2.5) [30], a sequential-coding system for psychotherapy sessions that was derived from the Sequential Code for Observing Process Exchanges (SCOPE) [31]. The SCOPE uses concurrent transcripts and audio recordings to divide and rate client and clinician speech; it was employed in Project PREMIR [6], a psychotherapy process study of 118 recordings of 13 Motivational Enhancement Therapy clinicians. The MISC 2.5 serves three purposes: parsing (unitizing) speech into codeable utterances (speech units), sequential coding of client and clinician utterances, and assignment of global ratings for clients and clinicians. Multiple versions of the program were tested and refined by trained MISC 2.5 coders before CACTI was employed in Project ELICIT.

### System Requirements

CACTI was developed and tested primarily on PC (Windows XP or higher), but was written in Java, and therefore is compatible with Macintosh, Linux, and Solaris systems. Software requirements include one of the above platforms, and Java Runtime Environment Version 1.6 or higher [32]. Hardware requirements are minimal, and include at least 1 MB of hard drive space, 64 MB of RAM, a basic sound card, an audio playback device (e.g., speakers or headphones), and a pointing device or keyboard.

### Features

CACTI is intended to facilitate the parsing and sequential coding of auditory behavioral interactions between two or more participants (such as those between clinician and client in a psychotherapy session), as well as to provide optional global ratings of features of those interactions. It does not rely upon transcripts, and instead allows coders to rate audio-recorded sessions electronically and in real time. The software offers the following features, which combine to provide CACTI with unique functionality.

#### Three modes

CACTI employs three modes that satisfy different functions: parsing continuous behavioral data from a WAV audio file into codeable utterances, sequentially coding previously parsed utterances, and assigning Likert-type global ratings. (See Figures S1, S2, and S3 for sample illustrations.) Those modes create output files with file extensions of.parse,.casaa, and.global, respectively; within these files, utterances are sequentially enumerated automatically, and codes are uniquely named and numbered by the user within the XML file (see operation manual, described below). All CACTI output files can be opened as tab-delimited text or spreadsheet files and then imported into statistical software for analysis.

#### Downloadable software package

The CACTI software package is a free 2 MB ZIP file (downloadable from http://code.google.com/p/ctcsu-player/downloads/list) that includes six components: the CACTI.jar archive file, UserConfiguration.xml file, images folder, operation manual [33], user-configuration guide [34], and the GetCASAA script [35] to convert CACTI output files to Sequential Data Interchange Standard (SDIS) [36] format. CACTI output files should be saved locally during parsing and coding, but sizeable WAV audio files may be stored on the local machine or on a shared local area network, as needed. A test dataset also is available from that software archive.

#### Customizable-coding-system feature

Although CACTI was designed originally for use with the MISC 2.5, it was intentionally built to be user-modifiable to accommodate other coding systems. Moreover, CACTI is free and open-source under the GNU General Public License, which means that anyone may use and modify it. These features facilitate CACTI’s utility for psychotherapy research–or audio-based content analysis more generally-rather than only within a motivational interviewing framework.

By default, the UserConfiguration.xml file is arranged for MISC 2.5 coding, but most elements of the three modes–including buttons, sub-menus, and keyboard shortcuts–are user-modifiable using XML. CACTI can be configured to accommodate any number of behavior codes or sliders, with limitations imposed only by the user’s screen resolution (e.g., 16 global ratings for 1280×800 pixels) and desired number of characters displayed in button names. For complex coding systems, behavior-code buttons may be expanded to become menus of sub-codes, which allows for the streamlined presentation of code–sub-code combinations. Detailed information about configuring the XML file is included within the software package in the operation manual and user configuration guide.

#### Automatic back up

To prevent data loss, each mode produces a real-time backup file that saves upon the placement of a new parse, behavior code, or global rating. This feature ensures that data are not lost if a CACTI session is interrupted.

#### Visual time line

The parsing and sequential-coding modes feature a visual timeline to help coders anticipate the ends and beginnings of utterances and ensure that codes are placed as intended. The time line appears as a series of colored rectangular boxes that move as a coder progresses through the audio file; start and end times, utterance enumeration, and assigned code names and numbers are displayed for the previous, current, and next utterances.

#### Playback and function buttons

In the parsing and sequential-coding modes, two panels of buttons control the playback and parsing or coding functions. Playback controls allow users to pause or resume audio, rewind, remove parses or codes, or extend the previous utterance. Function buttons allow users to designate beginnings and ends of parses or assign codes to pre-parsed utterances. These controls may be triggered by mouse or through keyboard shortcuts, which are intended to decrease coder reaction time, accommodate different user preferences, and reduce possible strain from repetitive motions.

#### Sliders

The global-coding mode includes multiple sliders that submit Likert-type global ratings of clients and clinicians, along with a text box for notes. The default (MISC 2.5) format uses seven five-point scales, but any number of scales and values may be specified.

#### Precise and comprehensive timing information

In CACTI, all parse and sequential-coding files are denoted to the hour, minute, second, and bit (see Example). Thus, utterances can be assigned as precisely as coder reaction time will allow, which can be beneficial in capturing events of brief duration or finely parsing audio information.

Example.


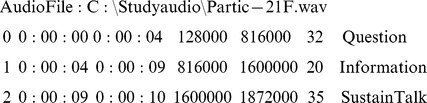


Note that the first column indicates the utterance number, the second and third the starting and ending times, the fourth and fifth the starting and ending byte numbers, and the sixth and seventh the numeric values and names of the codes assigned.

#### Ease of data analysis

Using the GetCASAA.m MATLAB script, users can summarize a set of.casaa files into a tab-delimited text file containing counts for each coding variable by session, an additional tab-delimited text file containing the cumulative duration of each coding variable by session (e.g., “talk time” for each speaker, or total time spent on task, rounded to the nearest second), as well as a Sequential Data Interchange Standard [36] file for the GSEQ application [37], organized by session. Count files can be imported into standard statistical packages, including R, SAS, or SPSS, for subsequent analysis. Lag-sequential analysis can be performed in GSEQ, estimating conditional probabilities and odds ratios for each possible transition, either pooled across a sample of sessions, or computed individually and exported for subsequent analysis.

## Results

### Advantages

CACTI shows many advantages over both non-computerized (i.e., transcript-based) coding methods and existing software.

#### Advantages over non-computerized coding methods

First, CACTI is more efficient than coding from transcripts, because it eliminates the lengthy and costly steps of transcribing sessions and manually entering data. Producing a clean transcript has taken our lab approximately 480–600 minutes per hour of audio, and other researchers have reported transcription times of up to 900 minutes per hour of audio [15], exclusive of parsing and coding time. In our experience, CACTI has required only 90 minutes per hour to parse and 75 minutes per hour to code; we estimate that the parent study, Project ELICIT, has saved at least 7500 work hours and upward of $60,000 by using CACTI instead of transcript-based methods. Second, the “talk time” for behaviors of theoretical interest can be calculated easily by adding the lengths of utterances, whereas previous methods required the manual use of a stopwatch, which was more difficult and error-prone. Other time-based calculations may be performed easily as well, by importing the output files into spreadsheets. Third, CACTI supports coding “on the fly” in real time, which, relative to using transcripts alongside audio recordings, might be more naturalistic, capture a greater scope of information, and avoid dividing coder attention. Fourth, the representation of interactions through transcripts has been criticized as potentially inaccurate and inherently “theory laden” [38], which CACTI avoids by referring coders only to the original audio source.

#### Advantages over other software

First, CACTI allows for the parsing, sequential coding, and global coding of audio files; these three features are not available together in any other software package that we could identify. CACTI’s ability to support the parsing of data prior to coding is a particular strength. As described in previous studies (e.g., [6], [10]), pre-parsing data can improve both interrater reliability and validity, because it ensures that coders are coding the same portion of the interaction. Second, CACTI is available to all users free of charge and intended for cross-platform use. These features support sharing and innovation, and remove some barriers to conducting research. Third, the software was designed to be easily modifiable by the user to match almost any audio-based coding system. Most aspects of the interface (e.g., rows, columns, titles, and values) can be changed in a simple XML file, and codes can be arranged hierarchically if needed. Fourth, the visual time line in the parsing and sequential-coding modes allows for greater precision than audio markers alone, which potentially improves interrater reliability.

#### Interrater reliability

Interrater reliability measures the degree of agreement among raters, and often is computed using intraclass correlations (ICCs) [39] in coding research. Per Cicchetti [40], ICCs above 0.60 represent “good” to “excellent” reliability, and have been considered acceptable in coding studies similar to Project ELICIT (e.g., [41]).

Project PREMIR [6], which performed sequential coding with concurrent transcripts and audio recordings, reported that ICCs for 10 of 14 summary ratings were above 0.75, which is within the “excellent” range described by Cicchetti [40]. The present study (ELICIT [29]), which used the CACTI program and a similar coding system, also achieved estimates of reliability in the “excellent” range on 10 of 14 of these same summary ratings. Both studies computed reliability using the ICC [3], [1] model [39]. To compare inter-rater reliability estimates between these two studies, ICCs were transformed to *z* scores using Fisher’s transformation and the effect size *q* [42] was computed. Results ranged from *q* = −0.40 (favoring PREMIR) to *q* = 0.69 (favoring ELICIT), with a mean of 0.11 (*SD = *.33). (See Table S1 for an overview.) These results suggest that coding with CACTI can produce comparable interrater reliabilities to those acquired via previous methods. We also have reported estimates of reliability for ELICIT using Krippendorff’s alpha [43], a measure of reliability than has been suggested for standard use in coding studies [44].

#### User acceptability and training needs

Although reliability is one measure of success, users’ subjective experiences of the software also are important in determining their satisfaction and their willingness to persist through the grueling and detail-intensive task of coding for a large, multi-year research project. Within our own lab, coders have reported that CACTI is useful, convenient, dependable, efficient, and easy to operate. In addition, we have found that training requirements are minimal, and offer substantial improvement over previous methods: Training four proficient MISC 2.5 coders to use CACTI reliably took approximately three hours per person in a combination of group and individual settings.

In summary, we have found that CACTI shows several advantages over both previous (i.e., transcript-based) sequential-coding methods and existing software. In addition, trained coders appear able to code standard psychotherapy-length verbal interactions (i.e., 45–60 minutes) and achieve similar interrater reliabilities as previous methods, while reporting less subjective difficulty than using transcripts, and substantially reducing expenditures of time and money. Training requirements were minimal, and users’ anecdotal reports indicated satisfaction with the product.

### Limitations

Although CACTI offers many advantages over other approaches to coding, we also have identified several limitations. Below we describe six such limitations and our recommendations for how best to circumvent them, when possible. First, files must be parsed before codes may be assigned to ensure that multiple coders rate the same segments of the interaction. Coding systems that do not pre-parse audio data before coding (e.g., MITI [45]) are incompatible with CACTI. Second, CACTI lacks the ability to denote overlapping speech or long content-free pauses through parsing, and transcripts–although cumbersome and expensive–may provide greater precision (e.g., [15]). To address these concerns, we created “decision rules” [30] to specify appropriate coder choices within such ambiguous situations: For example, coders should parse “beginnings, not ends” (i.e., make parses when the new speaker begins talking, rather than when the current speaker stops talking) and should parse out pauses of several seconds or more so that a “No Code” rating may be assigned later. We believe that this and other such decision rules assisted CACTI coders in obtaining acceptable interrater reliability. Third, only WAV digital-audio files may be used, and thus non-WAV digital files and analog recordings must be converted to the WAV format prior to use with CACTI. We accomplished this task quickly and easily using Audacity [46], a free and open-source program, along with a cassette player and 3.5 mm male–male stereo cable for converting analog recordings to digital files. Fourth, CACTI allows only for the coding of audio and does not support video or text formats. These capabilities cannot be modified in the UserConfiguration.xml file, but potentially could be added through significant additional programming in the original source code. Finally, technical support for CACTI is not currently available from the University of New Mexico Center on Alcoholism, Substance Abuse, and Addictions (UNM CASAA). However, a detailed user manual is included in the downloadable CACTI software package, and we anticipate that the original CACTI program and any future adaptations will be hosted on the UNM CASAA web site indefinitely.

## Discussion

The most obvious use of CACTI lies in the examination of motivational interviewing because the software was developed specifically to analyze that approach. Indeed, our data indicate that we have been able to obtain good reliability between raters for those process measures using this software, which is similar to previous results obtained with transcripts. Yet CACTI is atheoretical: By intent, it is a tool that can be modified to fit other treatment approaches–even those that do not employ specific theoretically derived techniques, but instead focus on more general factors thought to explain treatment success (e.g., [47]). The CACTI software potentially could serve as a framework for examining the process and content of any treatment for which definitions can be given to the elements that comprise it, with much less work than more traditional methods.

Future directions for the development of CACTI are apparent and exciting. First, adapting CACTI to include video would expand greatly its applications for research, particularly for describing instances of verbal behavior that cannot be captured through a purely auditory analysis (e.g., facial expressions, gestures, and other “body language”). This feature also would help CACTI compete with commercially available products, which could make behavioral research more financially feasible for academic or nonprofit research institutions. Second, we would welcome the addition of a mode to allow for the automatic randomization, ID masking, and multiple-coding assignment of study sessions, which would enable the automatic calculation of interrater reliability. Calculating interrater reliability instantaneously and continuously as a coding project progresses offers the potential for correcting coder drift in a targeted and efficient manner across the lifespan of a research project. Moreover, such a function would allow researchers to address demands for assessing reliability using only a portion of coders (e.g., those who coded the greatest quantity of sessions), or across different time points (e.g., calculating interrater reliability for every 100 sessions) when research studies are complex and lengthy. Finally, we recognize the limits of our current software in coding psychotherapeutic interactions that do not fit within a traditional dyad (i.e., client and clinician). For example, many therapy sessions include a significant other, and coding systems are now being developed to evaluate the manner in which concerned others might contribute to better (or worse) therapy outcomes (e.g., [48]). It is possible that CACTI could be modified to include significant others or even to evaluate group treatment formats, which would permit the study of mechanisms within those treatment configurations.

The CACTI software, although innovative in relieving coders of many tedious tasks, is not a panacea for the attention to detail that is required in the kind of meticulous research we discuss. We do not propose that any single technological tool will solve the many conceptual or methodological issues within the domain of psychotherapy process research. A tool, after all, still requires a user who makes decisions about how to employ it: A plow, no matter how marvelous, is not useful without a farmer. Similarly, CACTI only will be useful for researchers with imaginative and creative questions to ask about the nature of human verbal interactions. The contribution of this tool is to make such questions easier to answer, if not easier to imagine.

## Supporting Information

Figure S1 **CACTI parsing mode.**
(TIF)

Figure S2 **CACTI sequential-coding mode.**
(TIF)

Figure S3 **CACTI global-coding mode.**
(TIF)

Table S1 **Comparison of Interrater Reliability Estimates.**
(DOC)
