# Supplementary figures and images for: CACTI: Free, Open-Source Software for the Sequential Coding of Behavioral Interactions
Source: PLoS One. 2012 Jul 16;7(7):e39740. doi: 10.1371/journal.pone.0039740 (PMC3397966; doi:10.1371/journal.pone.0039740)

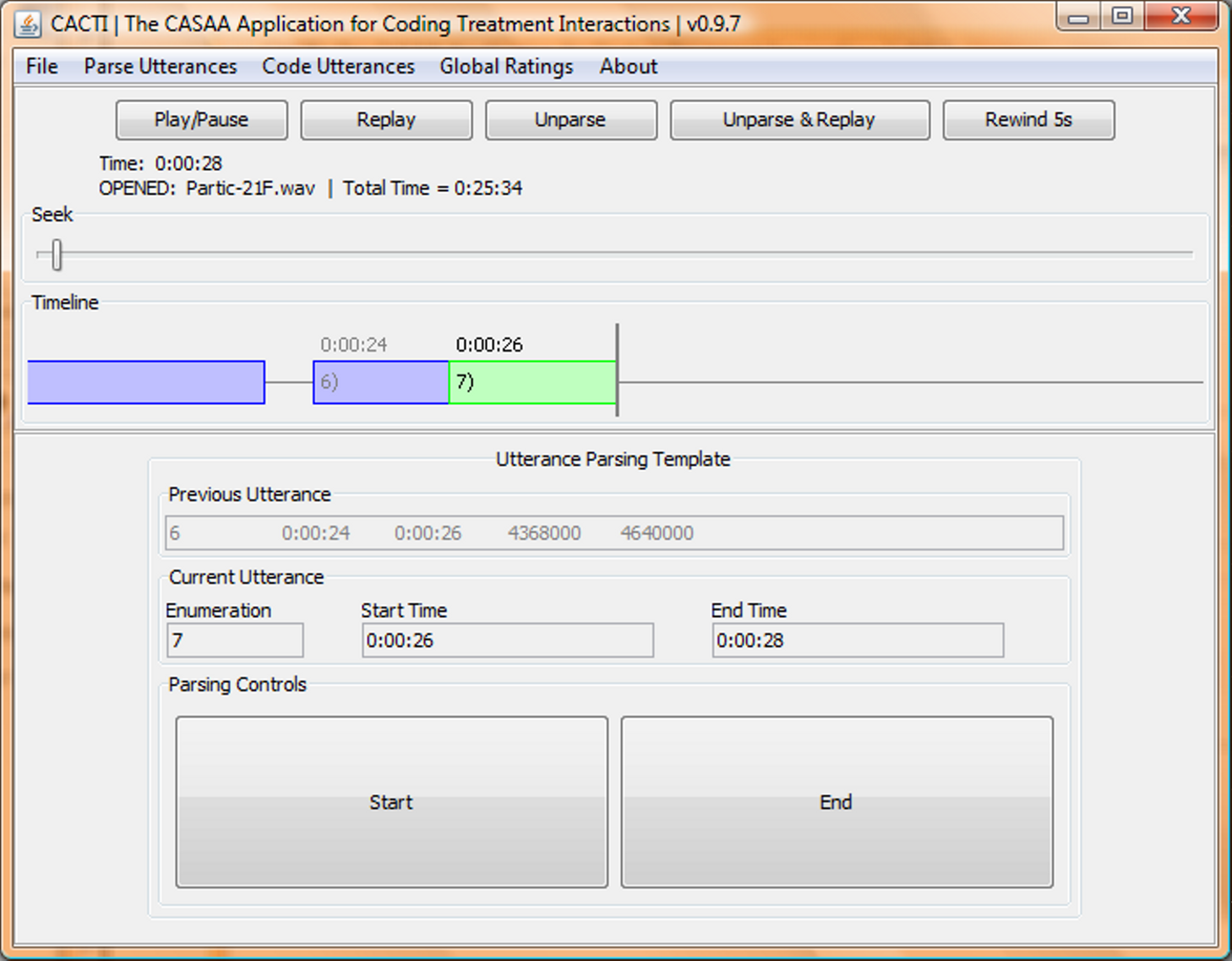

Supplement: Figure S1 — CACTI parsing mode. (TIF) [file pone.0039740.s001.tif]

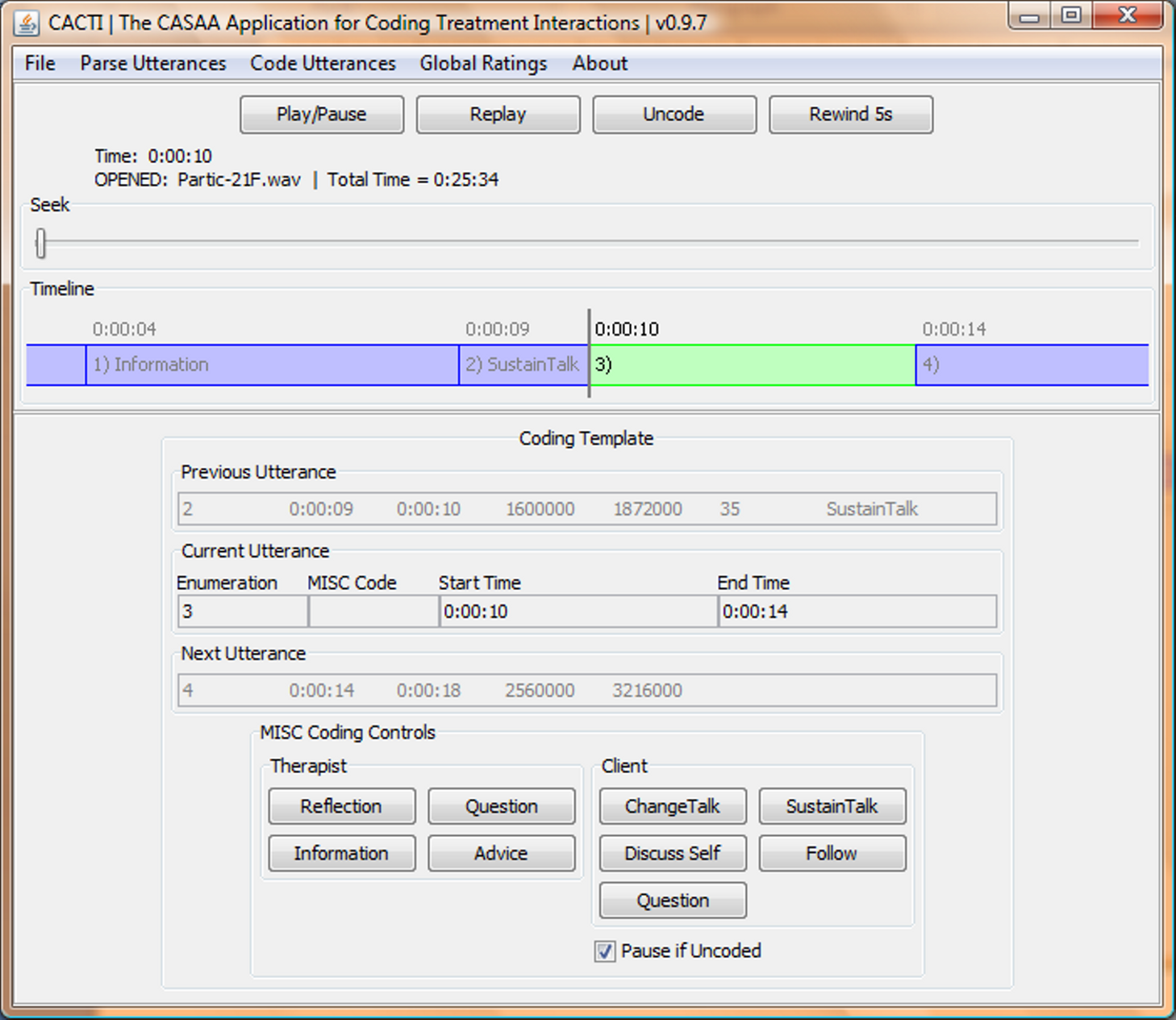

Supplement: Figure S2 — CACTI sequential-coding mode. (TIF) [file pone.0039740.s002.tif]

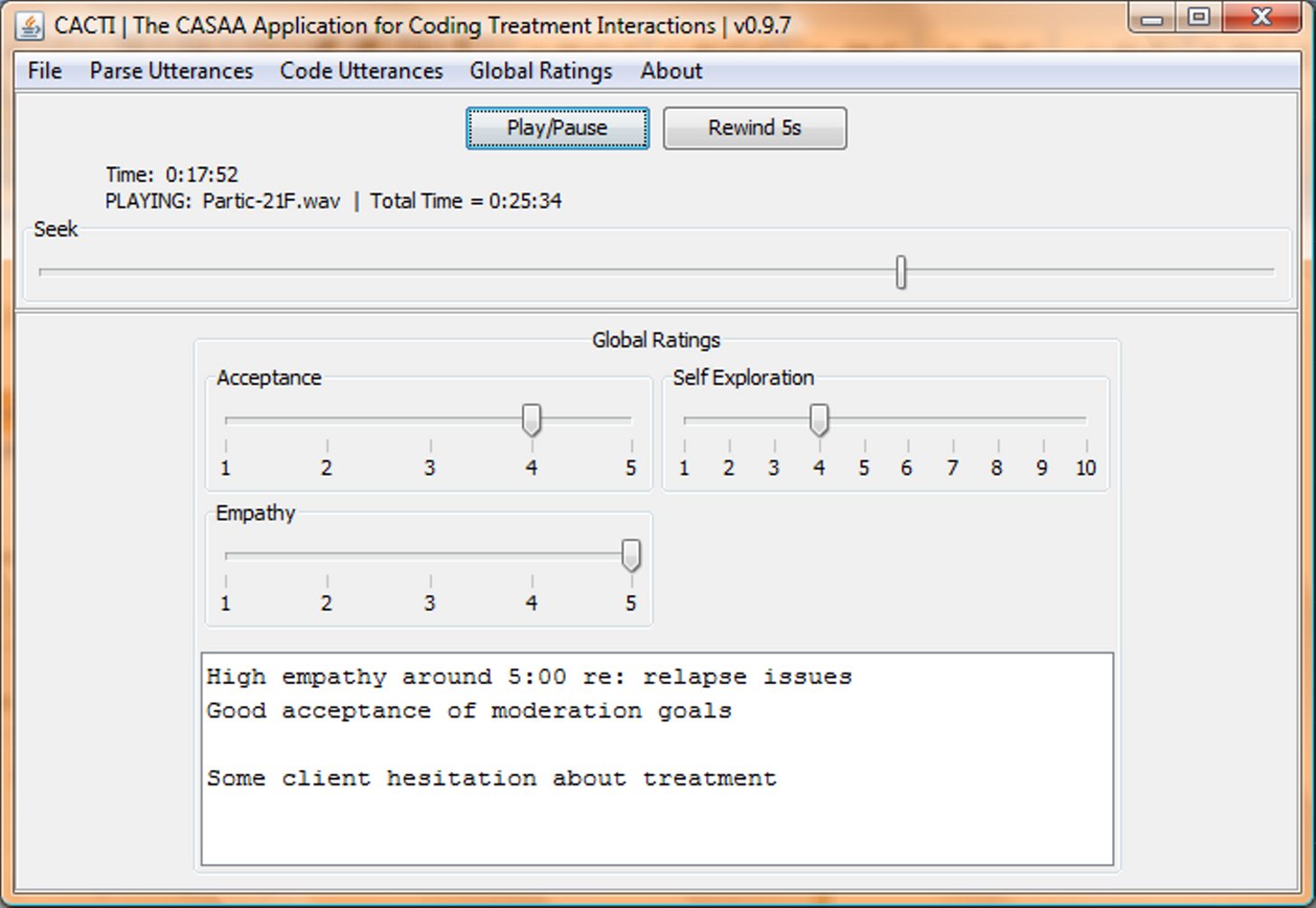

Supplement: Figure S3 — CACTI global-coding mode. (TIF) [file pone.0039740.s003.tif]
