# Supplementary material for: CACTI: Free, Open-Source Software for the Sequential Coding of Behavioral Interactions
Source: PLoS One. 2012 Jul 16;7(7):e39740. doi: 10.1371/journal.pone.0039740 (PMC3397966; doi:10.1371/journal.pone.0039740)
Supplement: Table S1 — Comparison of Interrater Reliability Estimates. (DOC) [file pone.0039740.s004.doc]

**Table**

*Table S1*

Comparison of Interrater Reliability Estimates

| Summary variable | PREMIR ICC | ELICIT ICC | *q* | ELICIT *α* |
| --- | --- | --- | --- | --- |
| CT | 0.88 | 0.93 | 0.28* | 0.79 |
| CCT | 0.86 | 0.90 | 0.18* | 0.56 |
| Follow/Neutral/Ask | 0.96 | 0.93 | -0.31** | 0.89 |
| MICO | 0.84 | 0.94 | 0.55*** | 0.92 |
| MIIN | 0.79 | 0.59 | -0.40** | 0.46 |
| Question | 0.98 | 0.99 | 0.30** | 0.98 |
| Closed Question | 0.98 | 0.97 | -0.24* | 0.94 |
| Open Question | 0.90 | 0.89 | -0.03 | 0.83 |
| Reflect | 0.92 | 0.95 | 0.27* | 0.92 |
| Reflect CT | 0.85 | 0.86 | 0.05 | 0.60 |
| Reflect CCT | 0.73 | 0.61 | -0.22* | 0.30 |
| Reflect Other | 0.56 | 0.87 | 0.69*** | 0.78 |
| Simple Reflection | 0.72 | 0.74 | 0.04 | 0.65 |
| Complex Reflection | 0.49 | 0.75 | 0.43** | 0.59 |

*Note*. PREMIR ICCs are replicated from [6]; *n* = 40, *k* = 3. ELICIT ICC *n* = 72, *k* = 6.
ICC = intraclass correlation; CT = change talk; CCT = counter-change talk.
* = small effect size; ** = medium effect size; *** = large effect size.
